# Returning genetic risk information for hereditary cancers to participants in a population-based cohort study in Japan

**DOI:** 10.1038/s10038-024-01314-w

**Published:** 2025-01-17

**Authors:** Kinuko Ohneda, Yoichi Suzuki, Yohei Hamanaka, Shu Tadaka, Muneaki Shimada, Junko Hasegawa-Minato, Masanobu Takahashi, Nobuo Fuse, Fuji Nagami, Hiroshi Kawame, Tomoko Kobayashi, Yumi Yamaguchi-Kabata, Kengo Kinoshita, Tomohiro Nakamura, Soichi Ogishima, Kazuki Kumada, Hisaaki Kudo, Shin-ichi Kuriyama, Yoko Izumi, Ritsuko Shimizu, Mikako Tochigi, Tokiwa Motonari, Hideki Tokunaga, Atsuo Kikuchi, Atsushi Masamune, Yoko Aoki, Chikashi Ishioka, Takanori Ishida, Masayuki Yamamoto

**Affiliations:** 1 Tohoku Medical Megabank Organization, Tohoku University, Sendai, Miyagi Japan; 2 Advanced Research Center for Innovations in Next-Generation Medicine, Tohoku University, Sendai, Miyagi Japan; 3 Department of Clinical Genetics, Ageo Central General Hospital, Ageo, Saitama Japan; 4 Department of Breast and Endocrine Surgical Oncology, Tohoku University Graduate School of Medicine, Sendai, Miyagi Japan; 5 Department of Gynecology and Obstetrics, Tohoku University Graduate School of Medicine, Sendai, Miyagi Japan; 6 Department of Clinical Oncology, Tohoku University Graduate School of Medicine, Sendai, Miyagi Japan; 7 Department of Clinical Genetics, Jikei University Hospital, Tokyo, Japan; 8 Department of Pediatrics, Tohoku University Graduate School of Medicine, Sendai, Miyagi Japan; 9 Graduate School of Information Sciences, Tohoku University, Sendai, Miyagi Japan; 10 International Research Institute of Disaster Science, Tohoku University, Sendai, Miyagi Japan; 11 Department of Molecular Hematology, Tohoku University Graduate School of Medicine, Sendai, Miyagi Japan; 12 Division of Obstetrics and Gynecology, Faculty of Medicine, Tohoku Medical and Pharmaceutical University, Sendai, Miyagi Japan; 13 Department of Rare Disease Genomics, Tohoku University Graduate School of Medicine, Sendai, Miyagi Japan; 14 Department of Gastroenterology, Tohoku University Graduate School of Medicine, Sendai, Miyagi Japan; 15 Department of Medical Genetics, Tohoku University Graduate School of Medicine, Sendai, Miyagi Japan

**Keywords:** Genetics research, Disease prevention, Psychology

## Abstract

Large-scale population cohort studies that collect genomic information are tasked with returning an assessment of genetic risk for hereditary cancers to participants. While several studies have applied to return identified genetic risks to participants, comprehensive surveys of participants’ understanding, feelings, and behaviors toward cancer risk remain to be conducted. Here, we report our experience and surveys of returning genetic risks to 100 carriers of pathogenic variants for hereditary cancers identified through whole genome sequencing of 50 000 individuals from the Tohoku Medical Megabank project, a population cohort study. The participants were carriers of pathogenic variants associated with either hereditary breast and ovarian cancer (*n* = 79, median age=41) or Lynch syndrome (*n* = 21, median age=62). Of these, 28% and 38% had a history of cancer, respectively. We provided information on cancer risk, heritability, and clinical actionability to the participants in person. The comprehension assessment revealed that the information was better understood by younger (under 60 years) females than by older males. Scores on the cancer worry scale were positively related to cancer experiences and general psychological distress. Seventy-one participants were followed up at Tohoku University Hospital; six females underwent risk-reducing surgery triggered by study participation and three were newly diagnosed with cancer during surveillance. Among first-degree relatives of hereditary breast and ovarian cancer carriers, participants most commonly shared the information with daughters. This study showed the benefits of returning genetic risks to the general population and will provide insights into returning genetic risks to asymptomatic pathogenic variant carriers in both clinical and research settings.

## Introduction

Pathogenic variant (PV) carriers of actionable hereditary diseases, such as hereditary breast and ovarian cancer (HBOC) and Lynch syndrome (LS) (ref. [1, 2]) have been identified in large-scale population cohort studies that have collected genomic information. PV carriers with early-onset cancer, multiple malignancies, or strong familial cancer history may be suspected of hereditary cancer risk and offered genetic testing. However, asymptomatic carriers often remain unaware of their genetic predisposition. Returning actionable genomic results to at-risk individuals could enable early intervention and disease prevention through medical surveillance. For instance, the Estonian National Biobank conducted a pilot study of returning genomic results to PV carriers of *BRCA 1/2* and other hereditary breast cancer-related genes (ref. [3, 4]). In the latter study, genotyping data were obtained from 66 161 participants, and 180 PV carriers were re-contacted for genetic counseling. Over 100 PV carriers participated in the study, and 100 of them visited clinical oncologists. Returning actionable genomic results to the population cohort study participants was also applied in The All of Us Research Program (ref. [5]), Mass General Brigham Biobank (ref. [6]), and US Department of Veterans Affairs Million Veteran Program (ref. [7]). There are many points to consider for the return of genomic results (ROGR) to research participants. For instance, the analytical and clinical validity of the results should be ensured, participants’ willingness to know genomic results should be confirmed in advance, and notification to at-risk relatives should be carefully considered (ref. [8]). While it is difficult to identify the best practices for ROGR to research participants that can be applied to all types of research, Global Alliance for Genomics and Health issued “2021 Policy on Clinically Actionable Genomic Research Results,” a global standard for ROGR to research participants (ref. [9]). The policy aims to set minimal standards and allows for appropriate customization by local and research projects. For European research projects, Vears et al. [10] recently provided a practical checklist for the return of genomic research results in the European context (ref. [10]). In June 2023, Japan enacted legislation aimed at the comprehensive and systematic promotion of personalized genomic medicine. In connection with this, The Japanese Association of Medical Sciences, The Japanese Medical Science Federation, and The Japanese Medical Association proposed the issues to be addressed to promote genomic medicine in March 2024. They pointed out that the improvement of genetic literacy in the general population is important and that medical genetics departments with clinical geneticists and genetic counselors should be universally distributed throughout medical institutes in Japan.

The Tohoku Medical Megabank (TMM) project is a large-scale population cohort study of over 150 000 residents of the Miyagi and Iwate prefectures in Japan (ref. [11, 12]). Several types of biospecimens, health information, and genomic and multi-omics data collected from participants have been incorporated into an integrated biobank that can be used by researchers (ref. [13, 14]). It also provides an open resource named jMorp (ref. [15]) (https://jmorp.megabank.tohoku.ac.jp/), which includes an allele frequency panel constructed from short-read whole genome sequencing (WGS) analysis. The TMM project aimed to promote personalized healthcare management in the general regional population. Therefore, at the time of enrollment, participants were informed that medically actionable genomic results would be returned to individuals who wished to know their genomic results. When WGS data collected from participants reached approximately 4000 individuals, a series of small-scale studies for ROGR began to assess possible benefits, validate the return process, and evaluate the psychological impacts on the participants with careful ethical considerations (ref. [16–18]). The participants were asked about their willingness to participate in the ROGR, and the information was provided in person. The first ROGR study focused on familial hypercholesteremia (ref. [16]), and actionable variants or genotype information regarding specific pharmacogenomic genes were returned to the participants to inform them of the potential risk of adverse drug reactions in the second study (ref. [17]). In the third study, we returned genomic results to six PV carriers of HBOC and referred them to Tohoku University Hospital (TUH) for medical surveillance (ref. [18]). The protocols established in these studies and participants’ favorable responses led us to expand the study to implement ROGR to the general population. Here, we report our experience and findings from surveys on returning genomic results to 100 carriers of PVs for HBOC and LS identified through WGS of 50,000 individuals from the TMM project.

## Materials and Methods

### Legal and ethical compliance

This study was conducted in accordance with the “Ethical Guidelines for Medical and Health Research Involving Human Subjects” presented by the Ministry of Education, Culture, Sports, Science and Technology, Ministry of Health, Labor and Welfare, and Ministry of Economy, Trade, and Industry, and was approved by the ToMMo Research Ethics Review Board (approval number: 2023-4-027).

### WGS analysis

The comprehensive procedures for WGS will be described elsewhere; here, we provide a concise overview of the core methodology. Genomic DNA was sourced from samples such as peripheral blood, saliva, and cord blood, and sequencing was performed using various platforms, including the Illumina HiSeq 2500, HiSeq X Five, NovaSeq 6000, MGI DNBSeq G400, and DNBSeq T7. Notably, Toshiba Corporation conducted sequencing on a HiSeq X Five. In contrast, specific NovaSeq sequencing tasks were carried out by Takara Bio Co. Ltd., iLac Inc., and HaploPharma Inc. Details of our informatics pipelines have been reported previously (ref. [15, 19]). Following GATK Best Practices (ref. [20]), raw data in FASTQ format is aligned to the GRCh38 reference genome sequence using BWA (ref. [21]) or BWA-mem2. After alignment, base quality score recalibration (BQSR) is applied, followed by SNV and INDEL calling using GATK HaplotypeCaller. Multi-sample joint calling is subsequently performed, and variant filtering is done with the GATK Variant Quality Score Recalibration (VQSR) tool. Post-filtering, various statistical metrics, such as allele frequencies, are calculated to ensure the quality of the WGS data. The resulting allele frequency information and quality metrics, including base count and mean coverage for each WGS sample, are publicly accessible on the jMorp database (ref. [15]).

### PV interpretation

PVs were identified from WGS data of 50,000 individuals who participated in the TMM study. *BRCA1* and *BRCA2* variant pathogenicity was anonymously interpreted as previously described (ref. [18]) using ClinVar on April 16, 2022, The Human Gene Mutation Database (HGMD) version 2022.1, and InterVar v2.2.2, a bioinformatics software for the clinical interpretation of genetic variants according to the ACMG-AMP 2015 guidelines. InterVar was run using the default option, in which 18 criteria (PVS1, PS1, PS4, PM1, PM2, PM4, PM5, PP2, PP3, PP5, BA1, BS1, BS2, BP1, BP3, BP4, BP6, and BP7) were used for variant interpretation. PV interpretation of the LS-related genes *MLH1*, *MSH2*, *MSH6*, and *PMS2* was performed using ClinVar, HGMD, and InterVar. Candidate variants to be returned to participants had to satisfy at least two of the followings: 1) being pathogenic or likely pathogenic in ClinVar, 2) harboring disease-causing mutation in HGMD, and 3) being interpreted as pathogenic or likely pathogenic by InterVar. These variants were then examined by utilizing the International Society for Gastrointestinal Hereditary Tumours and the Leiden Open Variation Database, and colon cancer-associated variants reported in more than one database were selected to be returned.

The PV-negative carriers were chosen from the study participants carrying neither non-synonymous, stop-gain, and splicing SNVs nor insertion/deletion variants under 5% allele frequency in both HBOC- and LS-related genes.

### Participants, protocol and contributing stakeholders

**Fig. 1:**
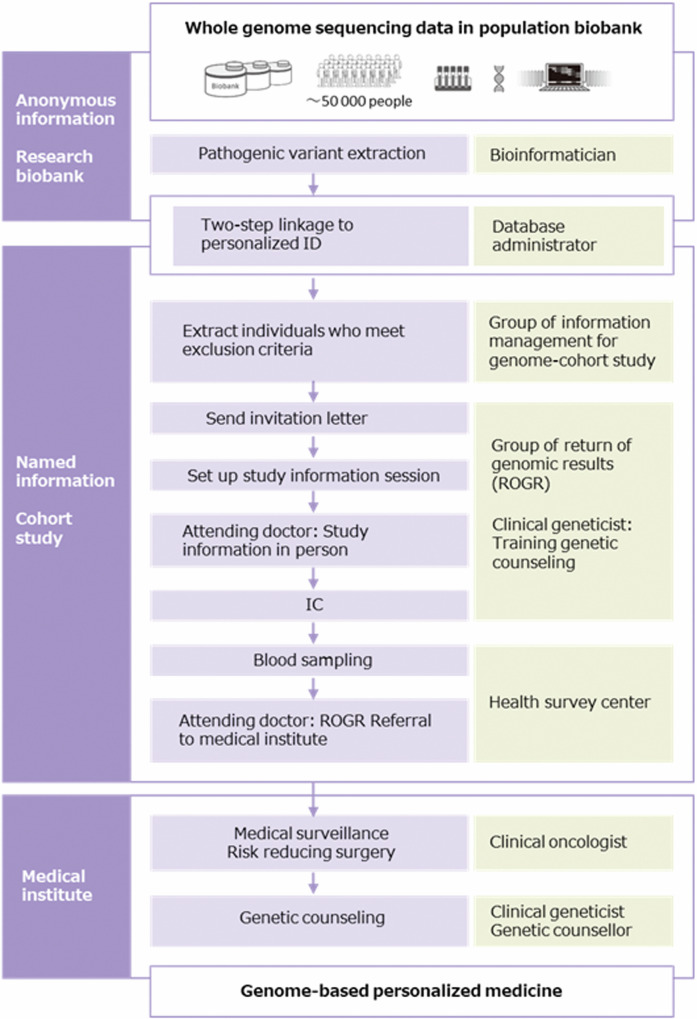
WGS data flow diagram. WGS data flow diagram of this study. The place where genomic information is handled (left column), processes of ROGR (middle column), and stakeholders in each process (right column) are shown

The ROGR process was conducted according to the protocol established in our previous study (ref. [18]) with slight modifications (Fig. 1). WGS data are anonymously stored in the TMM biobank under the control of our security policy (ref. [22]). Stakeholders who handle anonymous data are not allowed to access the personal information of PV carriers, and only a limited number of data administrators can link anonymous genomic data to individual real-name data (Fig. 1). After the individual real-name data was verified, the information management group for the genome cohort study assessed the personal information of PV carriers and excluded participants who met the exclusion criteria (deceased, under 20 years of age, previous ROGR study participants, and retracted consent from the TMM cohort study). An invitation letter asking about their willingness to know about their genomic results was sent to both PV-positive and PV-negative individuals, considering that the invitation itself did not indicate any notification of the genomic results. The ROGR was informed as important for health and medically actionable, whereas gene names or related diseases were not disclosed in the invitation letter. Next, in-person study information session was conducted for PV carriers who wanted to know their genomic results. Following the session, written IC was obtained from the participants, and blood sampling was conducted for single-site analysis to validate the genomic results. Single-site analysis was performed in an external clinical laboratory with a registration certificate. The validated genomic results were returned to the participants in person, and they were referred to TUH for medical countermeasures. When the attending physician or staff in the first session identified participants with insufficient comprehension, they relayed this information to the physician and staff conducting the second session. For participants with insufficient understanding, important information was rephrased using simpler terms and more time was allocated, as far as the schedule permitted. Additionally, with the participants’ consent, we invited family members to accompany them to the second session for additional support and understanding. Attending doctors, though not necessarily clinical geneticists, underwent training by a clinical geneticist and observed at least one ROGR session before conducting their own. For participants requesting hospital visits, doctors prepared referral documents, and staff assisted in scheduling appointments at TUH. The PV carriers of HBOC-related genes were referred to the Department of Breast Surgery and/or Gynecology, whereas those of LS-related genes were referred to the Clinical Oncology and/or Gynecology department.

### Questionnaire surveys (QSs)

A series of QSs was conducted to evaluate the participants’ psychological impact, cancer worry, comprehension level for the information, and attitudes toward their genomic risks. The surveys were conducted twice: after obtaining informed consent (IC) (QS1) and 12 months following the ROGR (QS2). The QS1 and QS2 included the Japanese version of the Kessler Psychological Distress Scale (K6) (ref. [23]), Japanese version of the Cancer Worry Scale (CWS-J), and a comprehension test. The CWS is a scale for evaluating cancer worry and its impact on mood and activities of daily living and is targeted at ages 16–84 years (ref. [24]). This scale can be used not only for cancer patients but also for non-cancer individuals. The CWS-J was developed and its validity and reliability were assessed by Drs. Otsuka and Fukumori, Tokushima University, and was used in this study with permission. It consists of 8 items, each with four choices: “rarely,” “occasionally,” “often,” and “always.”

### Statistical analysis

The IBM SPSS Statistics (version 29.0.1.0 [171]; IBM Japan, Tokyo, Japan) software package was used to perform statistical analyses. Spearman’s rank correlation coefficient was used to screen the correlation between demographic and observed factors, i.e., participant age and sex, syndrome type, comprehension test, CWS-J and K6 scores, cancer experience, and behavior of visiting the hospital. The significance of the differences in the results of each question item of the CWS-J between participants with and without a history of cancer was evaluated in 2×2 contingency tables using Fisher’s exact test. The results of answers were divided into two groups: group 1 (“rarely” and “occasionally”) and group 2 (“always” and “often”). The significance of the difference in comprehension levels was determined using a *t*-test. A paired *t*-test was used to evaluate the difference in means of K6 and CWS-J scores at two time points. The significance of the difference in the total CWS-J scores of four groups (K6: high/low and cancer experience: +/-) was determined using a one-way analysis of variance test with Turky-Kramer ad hoc test. The significance of the correlation of sex and generation with the shared status of genetic information with relatives (shared or unshared) was evaluated using logistic regression analysis. Generation was coded as follows: parent=0, sibling=1, and offspring=2. Sex was considered a categorical variable. *P* < 0.05 was considered significant.

## Results

### Recruitment of participants

**Fig. 2:**
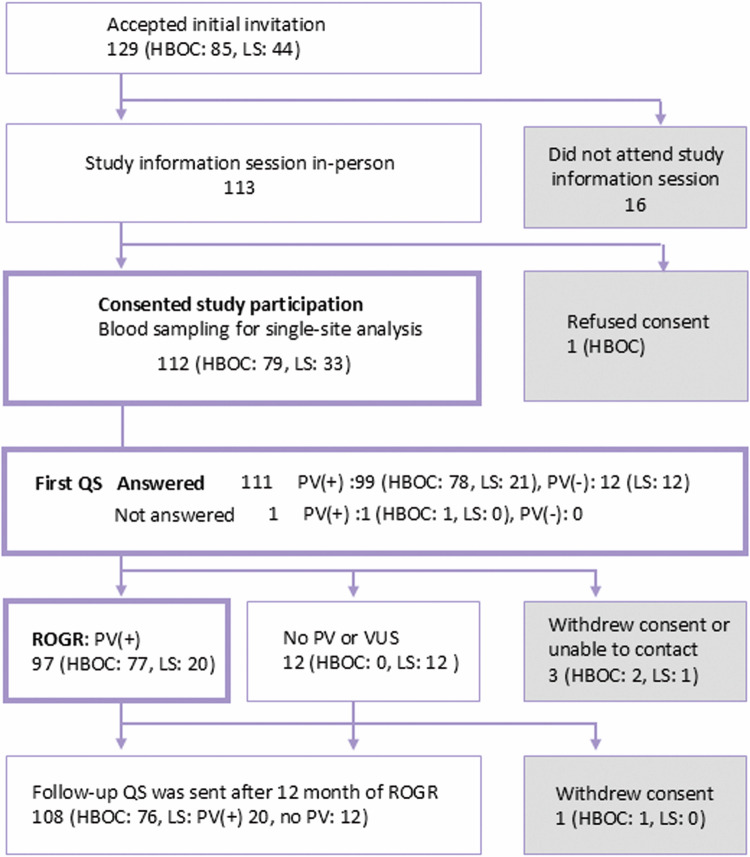
Overview of the study participants. The number of participants and their responses to the study process are presented. QS: questionnaire survey

Responses to recruitment are shown in Supplementary Table 1. Approximately two-thirds of non-PV carriers (477 of 756, 63.1%) wished to know their genomic results, even though they were not notified of the names of genes to be returned or of disease risks. The invitation letter was sent to 238 PV carriers (HBOC: 167, LS: 71). A reminder letter regarding the response was sent exclusively to PV carriers. After the second round of reminder letters, 129 PV carriers (HBOC: 85, LS: 44) accepted the invitation and a study information session was arranged for them at the nearest local assessment center (Supplementary Table 1). Of the 129 PV carriers who accepted the invitation, 113 attended the study information session in person and 112 consented to participate in the study (Fig. 2). The remaining 16 individuals who accepted the invitation were unable to attend the information session due to various personal reasons. One male *BRCA1* PV carrier refused to participate in the study despite having attended an information session. Blood sampling for single-site analysis and the first QS were conducted after IC was obtained. The results of the single-site analysis were verified with those of the WGS and were returned to the participants in person.

The results of non-PV carriers who accepted the invitation were sent by mail. We provided an outline of the study, including the technical limitations of WGS. We also provided a URL (https://www.megabank.tohoku.ac.jp/activity/localhealth/rogr/movie) of an educational video for non-PV carriers that we originally created and uploaded on social media.

### Individual variant and cancer incidence

The list of PVs and number of participants with HBOC and LS are shown in Supplementary Tables 2 and 3, respectively. There were 28 and 51 PV carriers of *BRCA1* and *BRCA2*, respectively. Among them, 11 *BRCA1* and *BRCA2* PV carriers had a history of cancer. Three PVs, *BRCA1* c.188 T > A, *BRCA2* c.5576_5579del TTAA, and *BRCA2* c.6592 C > T, which are frequently found in Japanese HBOC patients (ref. [25–27]), had more than 10 carriers (Supplementary Table 2). Five and nine PVs of *BRCA1* and *BRCA2*, respectively, were not identified in our previous dataset from 3 352 participants (3.5KJPNv2) (ref. [28]), in a report using samples from the Biobank Japan (7 051 breast cancer patients and 11 241 controls) (ref. [25]), in the HBOC registration system of Japan reported in 2018 (127 and 115 *BRCA1* and *BRCA2* PV carriers, respectively) (ref. [26]), and in Japanese ovarian cancer patients (ref. [27]). The number of PV carriers of *MLH1*, *MSH2*, *MSH6*, and *PMS2* were six, one, nine, and five, respectively. Eight patients had a history of cancer. There were three PV carriers for *MSH6* c.3226 C > T and ≤2 for other PVs (Supplementary Table 3). None of the PVs, except *MLH1* c.199 G > A, were reported in the variants in a previous study (ref. [29]) involving 2 501 Japanese cancer patients or in data from the Japanese Society of Cancer of the Colon and Rectum reported in 2017 (ref. [30]).

Although the results of single-site analysis of all *BRCA1* and *BRCA2* variant carriers matched those of WGS, there was a discrepancy between the single-site analysis and WGS in four variants of the 12 LS participants (Supplementary Fig. 1). Three individuals with the *MLH1* variant c.2080 G > T (p.E694*) had another variant in an adjacent nucleotide (c.2081 A > C) in the same allele, resulting in the missense variant c.2080_2081inv (p.E694S). This variant has been previously reported and annotated as VUS (ref. [31]). In addition, three PVs from nine participants were not identified in the single-site analysis. All of these were the variants in or adjacent to the homopolymer regions that were supposed to be challenging for WGS-based screening. We informed participants of the unanticipated results and the inherent limitations of WGS technology. Besides, we performed single-site analysis for the *MSH2 *c.942 + 3 A > T variant on 23 individuals using DNA samples stored in our biobank, rather than freshly-prepared blood samples. This was done because these individuals had accepted the invitation but were not scheduled for the study information session at that time. As none of these samples tested positive for the variant, we did not arrange an information session for these participants. Instead, their results were communicated via postal mail.

### Outline of the participants

The age group and sex of 112 PV carriers (HBOC: 79, LS: 33) who consented to participate in the study are shown in Table 1. At present, 108 individuals, including 12 non-PV carriers of LS, continue participation, whereas 4 participants (HBOC: 3, LS: 1) withdrew consent or discontinued contact. Thirty-seven males (HBOC: 25, LS: 12) and 75 females (HBOC: 54, LS: 21) participated in this study. Non-PV carriers of LS were 6 each for males and females. The median ages of HBOC and LS PV carriers were 41 and 62 years, respectively. There was a bimodal age distribution for both syndromes, and the difference of median ages between the syndromes was not statistically significant (*P* = 0.30, Mann-Whitney test). Most participants in their 40 s participated in the TMM Birth and Three-Generation Cohort Study (ref. [32]), whereas those in their 70 s participated in the TMM Community-Based Cohort Study (ref. [33]).

**Table 1:** Profiles of study participants

Age group	HBOC			LS		
	Male	Female	Total	Male	Female	Total
30-39	1	11	12	3(2)	6(1)	9(3)
40-49	6	26	32	2(2)	7(4)	9(6)
50-59	0	7	7	0(0)	0(0)	0(0)
60-69	7	3	10	2(2)	4(1)	6(3)
70-79	10	7	17	4(0)	3(0)	7(0)
80-	1	0	1	1(0)	1(0)	2(0)
**Total**	25	54	79	12(6)	21(6)	33(12)

The numbers in parentheses indicate PV(-) participants

### Participants’ comprehension levels and cancer worry

**Fig. 3:**
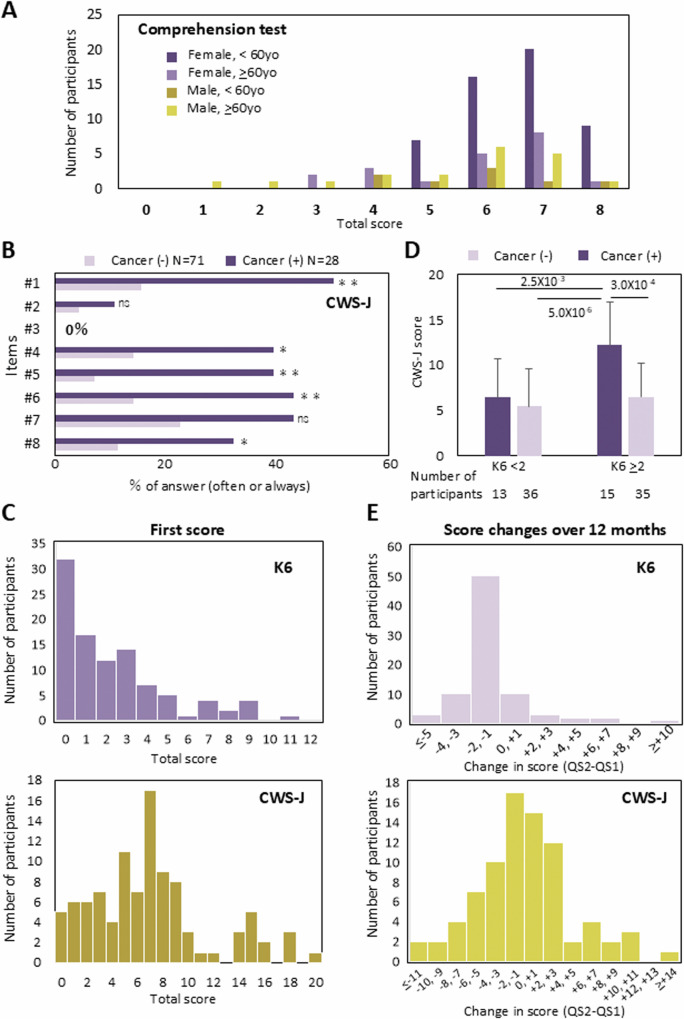
Related analysis of the comprehension test, CWS-J, K6, and cancer history. **A** Distribution of the comprehension test score. Participants were divided into two age groups ( < 60 and ≥ 60), and the scores are shown separately. **B** Frequency of the answers to items #1 to #8 of the CWS-J. Dark and light colored bars indicate answers from participants who had cancer (*n* = 28) and those who never had cancer (*n* = 71), respectively. *, **, ns: comparison of the frequency of answers “often” and “always” from the cancer experienced vs. never had cancer groups. *: *P* < 0.05, **: *P* < 0.01, ns: not significant. **C** Distribution of the K6 (upper panel) and CWS-J (lower panel) scores. Scores and number of participants are indicated. **D** Average CWS-J scores of the four participant groups. The participants were grouped according to the K6 score ( < or ≥2) and cancer experience. The number of participants in each group is shown at the bottom. **E** Distribution of the K6 (upper panel) and CWS-J (lower panel) score changes over 12 months. Differences in scores (QS2-QS1) are indicated

Participants’ comprehension levels of the study information and cancer worry were assessed in the first QS. As shown in Fig. 2, 99 PV carriers (HBOC: 78, LS: 21) responded to the QS, and the results were examined using correlation analysis. The items, choices, and ratios of correct answers on the comprehension test are shown in Supplementary Table 4 Although all items were communicated to the participants in person, the ratio of correct answers varied from 42 to 100%. The mean and standard deviation of the total scores of correct answers was 6.2 and 1.4, respectively. The total score on the comprehension test significantly correlated with participants’ age and sex (Fig. 3A). The mean score of participants aged <60 years was significantly greater than that of participants aged ≥60 years (6.48 ± 1.03 vs. 5.64 ± 1.68; *P* = 0.0026). When the mean score of younger (<60) and older (≥ 60) participants was compared in females and males separately, the difference was significant in females (6.60 ± 0.93 vs. 5.85 ± 1.50; *P* = 0.013) and non-significant in males (5.75 ± 1.39 vs. 5.42 ± 1.87; *P* = 0.659). The mean score of younger females was significantly higher than that of younger (*P* = 0.030) and older males (*P* = 7.7 × 10^-4^). None of the factors other than age and sex was significantly correlated with the comprehension test score.

The CWS-J items are listed in Supplementary Table 5. The answers for each item of the participants with a history of cancer (*n* = 28) and those who never had cancer (*n* = 71) are shown in Fig. 3B. In all items except item #3, the frequencies of the answer “often” or “always” were higher in participants with a history of cancer than in those who never had cancer. The differences were statistically significant for items #1, #4, #5, #6, and #8 (Fig. 3B). When the answers “rarely,” “occasionally,” “often” and “always” were scored as 0, 1, 2, and 3, respectively, the average total scores of participants with a history of cancer (mean ± standard deviation: 9.5 ± 5.3) were significantly greater than those of participants never had cancer (6.0 ± 3.9, *P* = 3.8 × 10^-4^).

In the first QS, the K6, which might be influenced by general psychological distress, was conducted in conjunction with the CWS-J. The histograms of the K6 and CWS-J scores are shown in Fig. 3C. The average score was 2.1 ± 2.4 and 2.5 ± 2.7 for with and without cancer history, respectively (*P* = 0.542). Although the average score was not affected by the cancer history, the individual K6 score was significantly correlated with the CWS-J score (correlation coefficient: 0.283, *P* = 0.0046). A variance analysis of the CWS-J with factors including age, sex, K6 score, and cancer history suggested a synergistic effect of the K6 score and cancer history (*P* = 0.017 for the interaction term). When participants were divided into four groups according to the K6 score and cancer experience, the mean CWS-J score was the highest in participants with a high K6 score and positive cancer history (Fig. 3D). Moreover, it is noteworthy that none of the examined factors—including disease type, age, sex, comprehension level, CWS-J score, K6 score, and cancer history—correlated with participants’ attitudes toward addressing their genomic risks at the hospital. We analyzed the scores of the comprehension test, CWS-J, and K6 from PV carriers of BRCA1/2 and MMR-related genes separately to test for any differences between these groups. Interestingly, the average K6 score was significantly higher in participants with HBOC compared to those with LS (2.67 ± 2.8 vs 1.19 ± 2.8, *P* = 0.022; t-test). However, the average CWS-J score was comparable between participants with HBOC and LS. Notably, the number of participants who had previously had cancer was higher in the LS group than in the HBOC group. Therefore, we surmise that the higher K6 scores in HBOC participants were not directly influenced by their cancer genetic risks. No significant difference was observed in the comprehension test scores between HBOC and LS participants.

We evaluated K6 and CWS-J scores at two time points: at the time of obtaining IC (first time point) and 12-month following the ROGR (second time point). Of 96 PV carriers who received the second QS, 81 individuals completed both assessments for K6 and CWS-J. The correlation coefficient between the first and second assessment were 0.758 (*P* = 2.7 × 10^-16^) for K6 and 0.525 (*P* = 4.7×10^-7^) for CWS-J. The means and standard deviations of the K6 scores were 2.37 ± 2.69 at the first time point and 2.47 ± 3.82 at the second time point, with a P-value of 0.72. For the CWS-J scores, the means and standard deviations were 7.44 ± 4.68 at the first time point and 7.04 ± 5.52 at the second time point, with a P-value of 0.467. Figure 3E shows a histogram of the difference in scores between the two time points for K6 and CWS-J. The highest columns were between −2 and −1 in both K6 and CWS-J. These results suggest that knowing their genetic results had little impact on participants’ mood and worry about cancer.

### Participant follow-up in medical institutes

**Fig. 4:**
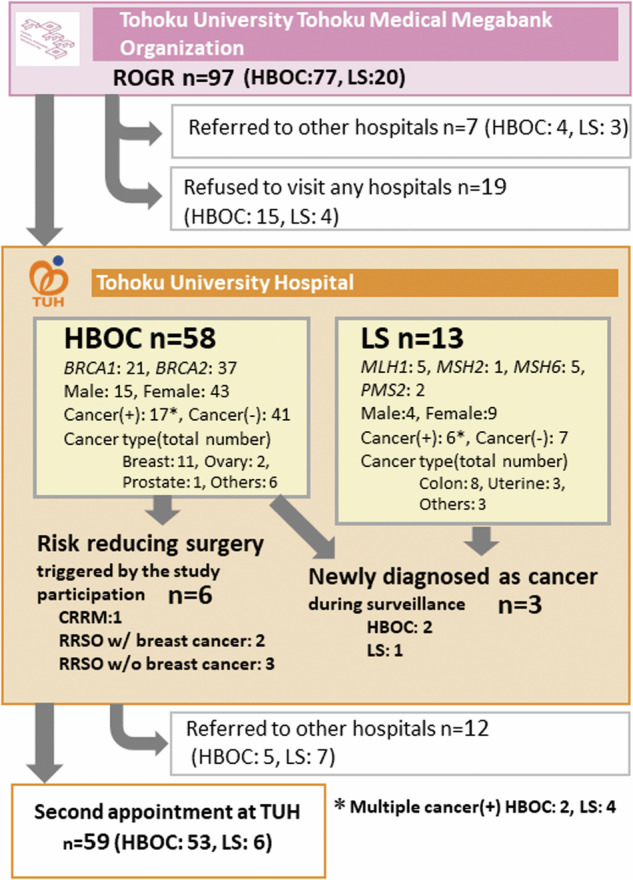
Clinical management of participants at Tohoku University Hospital. The number of participants who had the ROGR session and clinical management at Tohoku University Hospital is shown. The profiles of HBOC female participants who had breast and/or ovarian cancer are shown in Supplementary Table 4

All participants who underwent the ROGR session (HBOC:77, LS: 20) were advised to visit TUH. For four and three HBOC and LS participants, respectively, who had cancer and were followed up in other medical institutes, we directly created a patient referral document with genetic information for an attending doctor. Fifty-eight and thirteen HBOC and LS participants were referred to TUH, respectively (Fig. 4). Nineteen participants (HBOC: 15, LS: 4) refused to visit any medical institute. We asked participants to provide reasons for refusing hospital visits, allowing multiple responses. Six of them had once accepted to visit TUH but canceled thereafter or could not be contacted. Other participants stated several reasons for not visiting hospitals: nine of them raised personal reasons, such as old age, being busy with work, or raising children; six raised the burden of medical expenses; and five stated that TUH was too far from their place. Three participants who never had cancer believed that medical surveillance may not necessarily be valuable for themselves.

Of the 58 HBOC participants who visited TUH, 15 were males and 43 were females. There were 21 and 37 PV carriers of *BRCA1* and *BRCA2*, respectively. Most females visited both the Breast and Endocrine Surgical Oncology and Gynecology and Obstetrics departments on the same day. All males visited only the Breast and Endocrine Surgical Oncology department. Seventeen HBOC patients who visited TUH had various types of cancer, two of whom had multiple cancers. The profiles of 14 HBOC participants with breast and/or ovarian cancer are shown in Supplementary Table 6. Six breast cancer participants had contralateral risk reducing mastectomy. One participant (#4) decided to have a contralateral risk reducing mastectomy that was motivated by participating in this study. Similarly, two participants (#7 and #8) were motivated to undergo a risk reducing salpingo‒oophorectomy (RRSO) by the study participation. In addition, three participants who never had cancer underwent RRSO (Fig. 4). Two participants were diagnosed with cancer (#13: ovarian cancer and #14: right breast cancer) and received treatment. Of the 13 LS participants who visited TUH, four were male and nine were female. The numbers of PV carriers for *MLH1, MSH2, MSH6*, and *PMS2* were five, one, five, and two, respectively. Six of the thirteen participants had a history of cancer and four had multiple types of cancer (Fig. 4). None of the participants or their families were diagnosed with LS. One participant was diagnosed with colon cancer and underwent treatment. Twelve participants (HBOC: 6, LS: 6) were referred to the Department of Gastroenterology for surveillance. Following the first visit to TUH, 5 and 7 HBOC and LS participants, respectively, were referred to other hospitals, and 59 (HBOC: 53, LS: 6) had a second appointment at TUH (Fig. 4).

### Family history of cancer and sharing genomic results with relatives

**Fig. 5:**
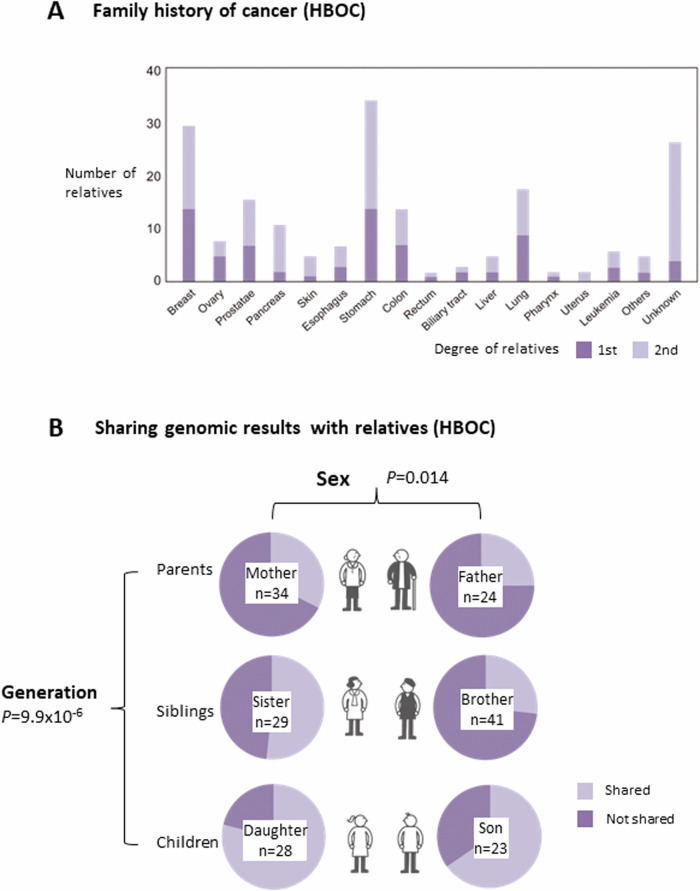
Family history of cancer and sharing genomic results with relatives in HBOC carriers. **A** Number of first- and second-degree relatives who had certain types of cancers as indicated. **B** Number of first-degree relatives with whom the participants shared or did not share genomic results. The first-degree relatives are shown separately according to sex and generation. n: the number of accessible relatives

The cancer types identified in the relatives of HBOC participants who visited TUH are shown in Fig. 5A. Stomach cancer was the most common type of cancer, followed by breast cancer. The 58 HBOC participants were asked whether information about genetic risk was shared with their family members (Fig. 5B). The results revealed that the offsprings were more informed about genetic risks by the participants than their parents (*P* = 9.9 × 10^-6^), and females were more informed than males (*P* = 0.014). However, only one female offspring decided to undergo genetic counseling and single-site analysis at the Department of Medical Genetics. She visited there 2 months after the first consultation at a clinical oncologist. According to the information from TUH, no other relatives underwent genetic counseling and single-site analysis at TUH.

## Discussion

We performed ROGR on the TMM research participants following the protocol established in our previous studies (ref. [16–18]). Regarding previous ROGR studies for HBOC variant carriers, the number of participants was limited to six, all of whom were over sixty years old (ref. [18]). The results from questionnaire surveys on the psychological impact of returning genomic results revealed large individual variations, making it difficult to draw conclusive findings. In contrast, this study conducted association analyses between participants’ characteristics and their scores from the comprehension test and cancer worry scales. Additionally, we observed that participants were more inclined to share genomic results with their children rather than their parents. These novel insights provide valuable information for both researchers and healthcare professionals involved in returning genomic results to participants and patients.

Our study led to the implementation of genome-based personalized risk reduction and early intervention strategies for cancer in a subset of participants. Notably, three participants who had never had cancer decided to undergo RRSO at their own expense. The participants who visited TUH continued follow-up medical surveillance either at TUH or other medical institutes, and three of them were diagnosed with cancer during surveillance. However, this study also revealed several issues, suggesting it may be premature to conclude that ROGR can be widely implemented in the general population. A major concern is the financial burden on participants who have never had cancer, as their medical expenses are not covered by health insurance, resulting in long-term costs. Additionally, the time required for medical surveillance can be a serious issue, especially for younger generations. Related to this, we found that approximately 20% of participants refused to seek medical attention despite being aware of their genomic cancer risks, citing time and financial burdens as reasons for not visiting hospitals. While these issues cannot be addressed by our research team alone, it is crucial to inform participants of such potential negative aspects. Consequently, we communicated the disadvantages of ROGR to study participants during the invitation process and at in-person information sessions. Furthermore, participants were given ample time to make decisions regarding study participation and medical surveillance.

Because we were aware of the importance of validation testing for ROGR, the analysis was entrusted to a clinical laboratory with a registration certificate for our studies (ref. [16–18]). The All of Us Research Program conducted a validation study and received an investigational device exemption from the United States Food and Drug Administration (ref. [34]). In fact, we encountered unexpected challenges in PV screening of DNA mismatch repair genes using WGS. Three PVs of mismatch repair genes from nine participants identified by WGS were false positives in the validation test, highlighting the necessity of confirmatory tests for ROGR. In line with this, Lincoln et al. [35] stated that approximately one in seven PVs associated with different hereditary diseases are complex variants identified using WGS. They classified these variants into six different types, including “low-complexity repeat-associated” variants like *MSH2 *c.942 + 3 A > T (ref. [35]). In addition, even though WGS sequencing was accurate, the current analysis pipeline did not consider any multi-nucleotide variants (MNVs), because of the high computational cost to detect all MNVs in addition to SNVs. However, the case of *MLH1* c.2080_2081inv, which overlapped with the PV c.2080 G > T, could be interpreted as an MNV. Therefore, to address this issue, we reimplemented Degalez’s approach (ref. [36]) to perform MNV detection applicable to large-scale WGS. Consequently, we construct an MNV panel for approximately 60 000 Japanese individuals named the “60KJPN NMV panel” and publicly released it on the jMorp database (https://jmorp.megabank.tohoku.ac.jp/). A summary table that focuses on MNVs located within exons in the 60KJPN-MNV panel is shown in Supplementary Table 7.

An analysis of the QSs showed that participants’ comprehension levels were correlated with age and sex. The scores were greater in females than in males, and the scores of participants younger than 60 years were higher than those of participants older than 60 years. However, the mean age of females was less than that of males (49.6 ± 13.5 vs. 63.8 ± 15.4; *P* = 2.0 × 10^-6^). Therefore, there is a possibility that both sex and age may be true causative factors of the score, or that either sex or age might be a confounding factor. When the scores of the younger and older groups were compared separately in females and males, the difference in females remained significant, suggesting that age may be a primary causative factor, at least in females.

In our previous ROGR studies (ref. [16–18]), we performed a longitudinal analysis of psychological scales, including the K6 (ref. [23]), Japanese edition of the profile of mood (ref. [37]), and Japanese-language version of the Impact of Event (ref. [38]), because awareness of genetic risk might cause significant psychological burden and/or posttraumatic disorders. This revealed that ROGR did not cause significant psychological distress, but large individual variations that tended to be stable throughout the study were observed (ref. [18]). In the present study, the K6 score and cancer experience synergistically affected the CWS scores, indicating that cancer worry might be largely influenced by participants’ depressive mood, which is represented by the K6 score. Several studies have examined cancer worry in breast cancer patients using the CWS (ref. [39, 40]). Consistent with our observations, the CWS scores were positively related to anxiety and depressive symptoms caused by factors unrelated to cancer (ref. [39]). Notably, neither comprehension level nor the CWS-J score affected participants’ behavior during hospital visits. Combined with the reasons raised by the participants who refused to visit the hospital, the behavior of taking measures against genetic risks might be affected by their current lifestyle, such as time and economic burdens. Therefore, further comprehensive analyses are required to address these issues.

Sharing genetic risk information in families is a key issue in disseminating genetic testing for at-risk carriers of hereditary cancer syndromes. A recent study reviewed the facilitators and barriers of cascade genetic testing in HBOC patients (ref. [41]). The present study revealed that HBOC risk information was shared more frequently with female relatives than with male relatives, which might be related to the characteristics of HBOC. Interestingly, significant differences were observed in this generation. The offsprings were shared genetic risk information the most, followed by siblings and parents in both sexes. Recently, Fukuzaki et al. [42] reported that Japanese hereditary cancer patients mostly shared genetic test results with their children aged>18 years (86.7%), followed by their siblings (73.6%) and parents (54.5%) (ref. [42]). Although their study and ours were conducted in different settings, it is interesting that similar results were obtained for Japanese participants irrespective of their cancer experience. Although the genomic results were shared by the proband, only one relative underwent immediate genetic counseling and testing. We considered the possibility that the information provided by the participants to their relatives was insufficient for them to make a decision. Alternatively, relatives might hesitate to visit medical institutes due to time and/or financial constraints, similar to some research participants who declined medical visits. Related to this issue, we asked the research participants about their relatives who knew the participants’ genomic results in the questionnaire survey performed 12 months after the ROGR. Sixty-four participants who shared their genomic results with relatives replied to the questionnaire survey conducted 12 months after the ROGR. The results revealed that 8 participants answered that there was at least one relative who was considering taking or had undergone genetic counseling at a medical institute. Given that only one relative actually visited the Department of Genetics at TUH, these results suggest that, while relatives understand the importance of genetic counseling, they may need time to decide on visiting medical institutes. Further follow-up is required to address this issue.

The value of population genetic biobanks is being increasingly recognized by researchers because patient-derived genomic information alone is insufficient to promote genome-based personalized medicine. Our results demonstrate that ROGR to participants who provide DNA samples to biobanks is feasible and beneficial. Since the current ROGR is mostly conducted for patients and their relatives, we established the ROGR protocol for the general population in this study. We also described the challenges that we faced during this study. We expect that our pioneering work will shed light on the expansion of ROGR in population genomic research.

## Supplementary information

Summary of supplementary information

Supplementary Figure 1. False-positive results of LS variants that appeared to be non-pathogenic in single-site analysis.

Supplementary Table 1. Number of individuals who responded to the study invitation.

Supplementary Table 2. List of PVs in BRCA1 and BRCA2 genes and incidence of cancer in the study participants.

Supplementary Table 3. List of PVs in MLH1, MSH2, MSH6, and PMS2 genes and incidence of cancer in the study participants.

Supplementary Table 4. Questions, choices, and correct answers of the comprehension test.

Supplementary Table 5. CWS-J items.

Supplementary Table 6. Profiles of HBOC female participants who had breast and/or ovarian cancer.

Supplementary Table 7. Summary of the changes of gene annotations in MNVs calls for the 60KJPN panel.
