# Supplementary material for: Returning genetic risk information for hereditary cancers to participants in a population-based cohort study in Japan
Source: J Hum Genet. 2025 Jan 17;70(3):147–57. doi: 10.1038/s10038-024-01314-w (PMC11802448; doi:10.1038/s10038-024-01314-w)
Supplement: Supplementary file 1 — Summary of supplementary information [file 10038_2024_1314_MOESM1_ESM.docx]

**Summary of supplementary information**

**Supplementary Figure 1. False-positive results of LS variants that appeared to be non-pathogenic in single-site analysis.**

Four pathogenic variants that were identified in 12 LS participants by WGS appeared to be non-pathogenic by single-site analysis. Variant information and results of single-site analysis are shown.

**Supplementary Table 1.** **Number of individuals who responded to the study invitation.**

Invitation for the study was sent by postal mail to both PV carriers (HBOC: N=167, LS: N=71) and no carriers (N=756). The number and percentage of individuals who accepted, refused, and did not respond to the invitation in each group is shown.

**Supplementary Table 2.** **List of PVs in** ***BRCA1* and *BRCA2* genes and incidence of cancer in the study participants**

Information of PVs in *BRCA1* and *BRCA2* genes, number of PV carriers and incidence of cancer in the study participants are shown.

**Supplementary Table 3. List of PVs in** ***MLH1*, *MSH2*, *MSH6*, and *PMS2* genes and incidence of cancer in the study participants**

Information of PVs in *MLH1, MSH2, MSH6*, and *PMS2* genes, number of PV carriers and incidence of cancer in the study participants are shown.

**Supplementary Table 4. Questions, choices, and correct answers of the comprehension test**

The comprehension test was conducted in the first QS and 99 of 100 PV carriers answered the QS. Percentage of correct answer for each question is shown in the right column.

**Supplementary Table 5. CWS-J items**

Items of the cancer worry scale adapted by Douma et al^20^ are shown. The Japanese-translated version of CWS (CWS-J) was examined in the first QS.

**Supplementary Table 6.　Profiles of HBOC female participants who had breast and/or ovarian cancer and are followed up at Tohoku University Hospital**

Profiles of 14 HBOC female participants who had breast and/or ovarian cancer and are followed up at Tohoku University Hospital are shown. Three participants who underwent risk reducing surgery were motivated by the study participants, and 2 participants were newly diagnosed with cancer.

**Supplementary Table 7. Summary of the changes of gene annotations in MNVs calls for the 60KJPN panel.**

Gene annotations were performed using SnpEff version 4.3t and the RefSeq Genome version 110 gene model. Only the MANE Select or RefSeq Select transcripts from the RefSeq Genome model were included. The If a gene overlaps with another, the same MNV is counted multiple times. When counting the number of MNVs based on the impact change, the distribution is as follows: unchanged: 215,557 MNVs (67.9%), up: 99,670 MNVs (31.4%), down: 1,845 MNVs (0.5%).
