# Supplementary figures and images for: Returning genetic risk information for hereditary cancers to participants in a population-based cohort study in Japan
Source: J Hum Genet. 2025 Jan 17;70(3):147–57. doi: 10.1038/s10038-024-01314-w (PMC11802448; doi:10.1038/s10038-024-01314-w)

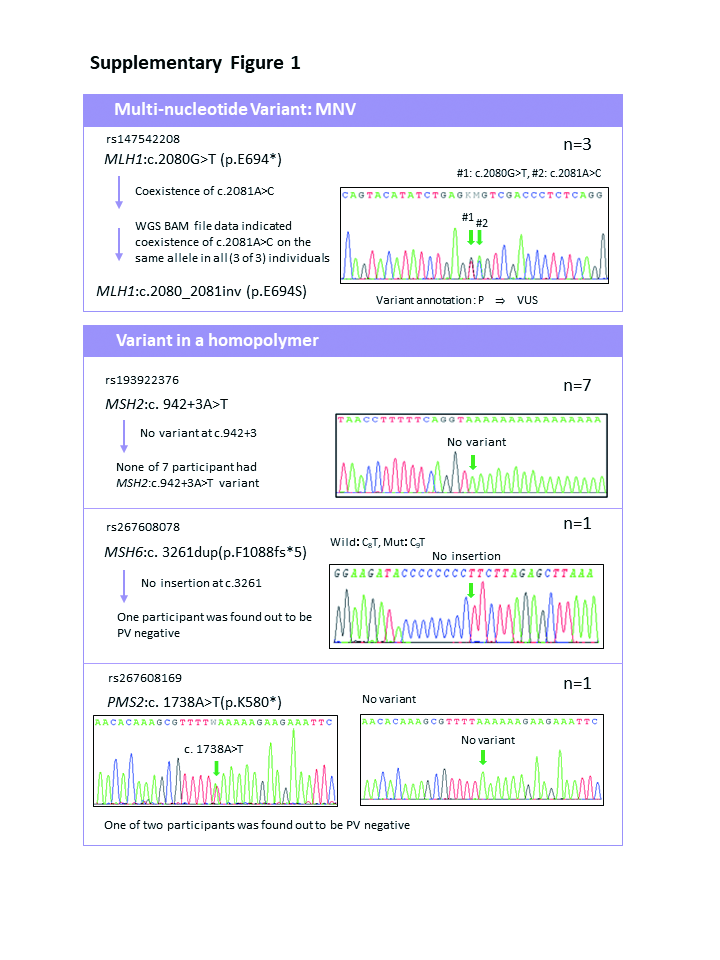

Supplement: Supplementary file 2 — Supplementary Figure 1. False-positive results of LS variants that appeared to be non-pathogenic in single-site analysis. [file 10038_2024_1314_MOESM2_ESM.tif]
